# Two-Step Polishing Technique for Flat and Smooth Copper Substrates by Electrochemical and Chemical Etching

**DOI:** 10.3390/mi17040466

**Published:** 2026-04-12

**Authors:** Ke Wang, Xinghua Chen, Boju Hou, Peng Xu, Yufei Li, Xutong Liu, Huirong Shi, Ming Zhang, Hongding Wang

**Affiliations:** 1 School of Mechanical Engineering, Lanzhou Jiaotong University, Lanzhou 730070, China; 17867289713@163.com (X.C.); 15839771080@163.com (P.X.); 18809498900@163.com (Y.L.); shrz98@aliyun.com (H.S.); zhm@mail.lzjtu.cn (M.Z.); wanghongding@gmail.com (H.W.); 2 Key Laboratory of Precision, Special Processing and Micro Manufacturing Technology of the Ministry of Education, Dalian University of Technology, Dalian 116024, China

**Keywords:** electrochemical etching, chemical etching, surface roughness, shape accuracy, mid-spatial-frequency

## Abstract

Methods of single-point diamond turning and chemical mechanical polishing can achieve an ultra-flat substrate. However, these methods which rely on mechanical interactions to achieve material removal can easily lead to defects such as abrasive embedding and scratches on the surface. In addition, for low-rigidity and thin-plate workpieces, clamping deformation and force deformation are critical factors affecting the machining accuracy. This paper proposes a two-step polishing chain that uses controllable electrochemical and chemical etching to correct the shape error of the workpiece. With the optimized parameters, the jet electrochemical machining (Jet-ECM), which uses the electrochemical etching mechanism, is applied to the computer-controlled optical surfacing (CCOS) to achieve the rapid convergence of the shape accuracy. In addition, electrogenerated chemical polishing (EGCP) is implemented as a follow-up process which uses the mechanism of diffusion-controlled chemical etching to reduce the mid-spatial-frequency (MSF) error caused by the computer-controlled optical surfacing. Based on this two-step polishing chain and the self-developed devices, the peak-to-valley (PV) value of the *φ* 50 mm workpiece (valid dimensions = 90% of the central region) is reduced from 2.678 μm to 0.384 μm. This study has great implications for further understanding the mechanism of Jet-ECM and EGCP, which expands the applications of stress-free polishing to solve the processing problems of the low-rigidity workpiece.

## 1. Introduction

Due to the excellent characteristics of thermal conductivity and electrical conductivity [1], the need for flat and smooth copper (Cu) substrates is increasing. For example, Cu planes are widely used as the growth substrates of functional crystal material [2], laser reflectors in CO_2_ laser systems [3], the substrates of molecular diffusion welding [4,5], and precision physical experiments [6,7]. Both the surface roughness and the shape accuracy of the substrates have important influences on the performances of the devices [8]. Therefore, there is a critical need for ultra-flat Cu surfaces which promise the performance of the devices.

Single-point diamond turning is a precision processing method to obtain an ultra-flat surface. However, the wear of the tool during the processing process would change the geometric accuracy of the tool, which reduces the surface shape accuracy of the workpiece significantly [9,10]. In addition, low-rigidity workpieces easily deform due to the cutting force, cutting temperature, residual stresses, clamping, and machining chatter. Such deformation significantly reduces the machining accuracy and surface quality [11]. Chemical mechanical polishing (CMP) is another commonly used precision processing method which removes the material through the synergy of chemical reactions and mechanical interactions. Yang et al. [12] used double-sided polishing which enables simultaneous material removal from both surfaces to weaken residual stress deformation and improve flatness. Wu [7] proposed a combined machining technology for thin copper substrates, including electrochemical lapping, chemical mechanical lapping, and low-pressure electrochemical mechanical polishing. A Φ100 mm × 3 mm copper substrate achieves PV 2 μm and *S*a 4.2 nm. However, because of the hard particles in the slurry such as SiO_2_ particles and Al_2_O_3_ particles, the surface defects such as abrasive embedding and scratches are generated on the surface, which affect the performances of the workpieces [13].

The stress-free machining method is the key to eliminating the influences of mechanical interaction, which does not rely on mechanical interactions and abrasives to realize the material removal. Therefore, the defects and deformations caused by mechanical force can be avoided, which is helpful to obtain a substrate with high shape accuracy. The commonly used stress-free methods are chemical polishing (CP) and electrochemical polishing (ECP). CP and ECP use the mechanism of viscous film to improve the surface quality. However, the viscous film shape changes with the workpiece shape which means the viscous film cannot correct the shape error of the workpiece [14,15,16]. Wet etching is a stress-free method which achieves material removal by chemical or electrochemical actions in the electrolyte. During the etching process, the workpiece does not need to contact any tool, so the cutting force, the cutting heat, the stress deformation, and mechanical defects are avoided [17]. In addition, there is no tool deformation in the processing process and the workpiece does not need to be clamped, which is helpful to achieve an ultra-flat Cu surface.

Jet electrochemical machining (Jet-ECM) is a non-traditional method which belongs to wet etching. The mechanism of Jet-ECM is electrochemical etching, which uses the hydraulic jump phenomenon of the electrolyte to constrain the territory of electrochemical effects to limit the material removal. Wang et al. [18] used Jet-ECM to fabricate the Cu substrate by superimposing the nozzle translating paths. The nozzle (cathode) in Jet-ECM is equivalent to a tool, and the workpiece (anode) under the nozzle is removed by anodic dissolution. The surface peak-to-valley (PV) value of the workpiece which indicates the surface shape error of the Cu is reduced to 1.7 μm. However, the mid-spatial-frequency (MSF) error generated after the processing limited the improvement of the workpiece shape accuracy [19]. Qiu et al. [20] proposed a predictive model for MSF errors to correlate the polishing process parameters. The results show that MSF errors decrease with increasing velocity ratio and decreasing step interval. Zhong et al. [21] pointed out that regular processing paths easily induce MSF errors, while random path superposition with random directionality offers a more effective solution for suppressing MSF errors. However, constrained by machine tool performance, the step interval cannot be infinitely reduced, and multiple iterations lead to low efficiency. Zhou et al. [22,23,24,25] proposed a novel stress-free polishing method named electrogenerated chemical polishing (EGCP) which is based on diffusion-controlled chemical etching to realize the material removal. The generation of the etching agent is controlled by the working electrode (WE) through electrochemical actions. The mass transfer of the ions in EGCP is controlled by diffusion, so the convex point of the workpiece has a higher concentration of the etching agent than the concave point, which leads to a higher material removal rate (MRR). However, the MRR is only hundreds of nanometers per hour, which is too low to correct the shape errors with several microns. Additionally, to obtain an ultra-flat surface, it is necessary to prepare an ultra-flat WE whose shape accuracy is much higher than that of the workpiece, which increases the cost and limits the application of EGCP.

In this study, a two-step polishing process chain was proposed which utilized controllable electrochemical and chemical etching to correct the shape error to achieve an ultra-flat Cu substrate efficiently. With the optimized parameters, the Jet-ECM was applied to the computer-controlled optical surfacing (CCOS) to achieve the convergence of the shape error with high efficiency. EGCP was implemented as a follow-up process to reduce the MSF error caused by CCOS. Through full-diameter chemical etching in EGCP, the MSF error was reduced. With the self-developed devices, an ultra-flat Cu substrate was finally achieved.

## 2. Mechanism and Model

### 2.1. Improve the Stability and Surface Quality of the Jet-ECM

Improving the surface quality of Jet-ECM is an important prerequisite for its application to CCOS. Existing studies have shown that Jet-ECM can machine the workpiece without mechanical defects and mechanical residual stress effectively. However, there are still shortcomings in reducing the surface roughness and improving the stability [8]. The mechanism of the Jet-ECM is anodic dissolution; for polycrystalline metals, different crystal planes have different etching rates which leads to uneven etching at grain boundaries [26,27]. Only when the viscous film, which has a larger resistance than the electrolyte, is formed can the uneven etching caused by the different crystal planes be avoided. The formation of the viscous film is beneficial for improving surface quality and stability.

**Figure 1:**
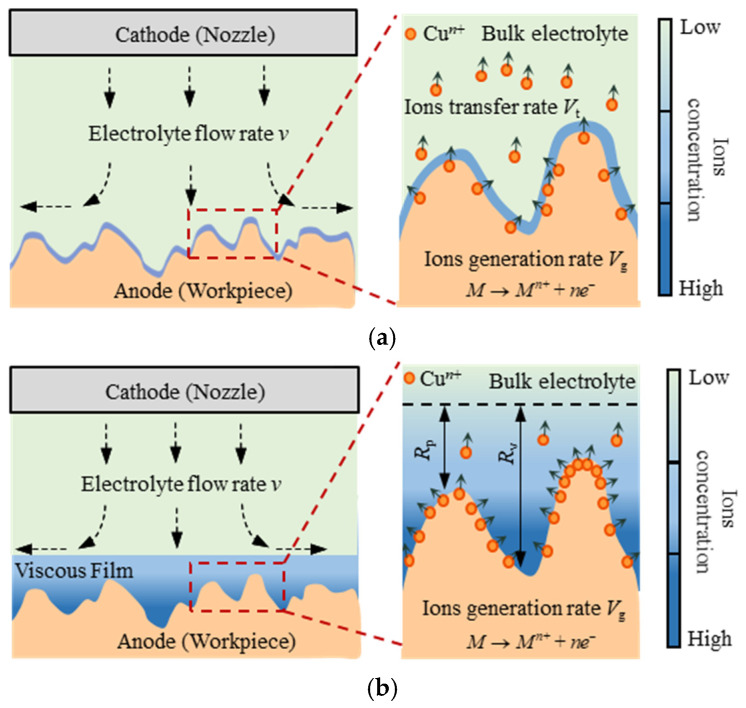
Schematic diagram of the viscous film in Jet-ECM. (**a**) *V*_g_ ≤ *V*_t_; (**b**) *V*_g_ > *V*_t_.

Existing studies showed that Cu atoms can be changed to Cu ions through reactions in the electrolyte [18].

(1) $$\mathrm{Cu}\to {\mathrm{Cu}}^{+}{+e}^{-}$$

(2) $${\mathrm{Cu}}^{+}\to {\mathrm{Cu}}^{2+}{+e}^{-}$$

with the generation of the Cu ions, some of the Cu ions transfer to the bulk solution through diffusion and convection. Other Cu ions are concentrated and bonded with anions to form a viscous film. The viscous film adheres to the workpiece surface, which has a higher impedance than the electrolyte [28,29]. The generation of the viscous film is not only related to the generation speed of the cations but also related to the mass transfer speed of the cations to the bulk solution. As shown in Figure 1a, when the mass transfer rate of cations (*V*_t_) is greater than or equal to the generation rate (*V*_g_), the viscous film cannot be formed. When the generation rate of cations (*V*_g_) is much greater than the mass transfer rate (*V*_t_), more and more cations accumulate on the workpiece surface to form a viscous film, as shown in Figure 1b. According to Faraday’s law, increasing the voltage can increase the current density, which is beneficial for increasing the cations. Additionally, increasing the duty ratio can generate more cations, which is an effective way to promote the formation of the viscous film. Meanwhile, the mass transfer rate of the cations to the bulk solution is affected by diffusion and convection, as shown in Equation (3).

(3) $$J=-D\nabla C_{M}+\nu_{L}C_{M}$$

where *C*_M_ is the concentration of the ions M and *D* is the diffusion coefficient of M. *ν*_L_ is the convection rate of M, and *J* is the total mass transfer flux of M.

According to the Stokes–Einstein equation [30], the diffusion coefficient *D* is inversely proportional to the viscosity of the electrolyte, as shown in Equation (4).

(4) $$D=\frac{kT}{6\pi \eta \gamma }$$

where *T* is the absolute temperature, *ϒ* is the radius of the ion, *η* is the viscosity of the electrolyte, and *k* is the Boltzmann constant.

According to Equation (4), when the viscosity of the electrolyte is low, the diffusion rate is fast, which leads to the formation of the viscous film with relative difficulty. With the increase in the electrolyte viscosity, the diffusion rate of ions decreases, which is conducive to the formation of the viscous film. In addition, in Jet-ECM, the electrolyte is ejected onto the workpiece surface from the nozzle at a high speed and the electrolyte flows at a high speed. The formation of the viscous film is affected by convection. According to Equation (3), reducing the flow rate of the electrolyte is conducive to the formation of the viscous film.

In this study, a specific electrolyte with a high viscosity is used. The proper parameters of voltages, duty ratios, and electrolyte flow rates promote the formation of the viscous film on the workpiece surface in Jet-ECM. After the formation of the viscous film, the surface quality and the stability of the Jet-ECM can be significantly improved.

### 2.2. The Dynamic Etching Analysis of EGCP

The material removal mechanism of EGCP is diffusion-controlled chemical etching and the chemical etching agents in EGCP are generated by electrochemical reactions on the working electrode (WE) surface. In EGCP, the mass transfer of the ions in the electrolyte is controlled by diffusion, which realizes the flattening of the workpiece. First, the intermediary (Fe^2+^ ions) around the WE surface can be oxidized to the etching agent (Fe^3+^ ions) through electrochemical reactions. At this time, the concentration of Fe^3+^ ions near the WE surface is much higher than the concentration of Fe^3+^ ions near the workpiece surface, resulting in a concentration difference. Driven by the concentration difference, the Fe^3+^ ions near the WE surface diffuse to the workpiece surface. Due to the high oxidation ability of Fe^3+^ ions, when the Fe^3+^ ions arrive at the workpiece surface, the Fe^3+^ ions react with Cu atoms to form Fe^2+^ ions and Cu^2+^ ions. The regenerated Fe^2+^ ions diffuse back to the WE surface and convert to Fe^3+^ ions again. During the whole process, the distance between the workpiece convex point and the WE surface is smaller than the distance between the workpiece concave point and the WE surface, so the concentration of Fe^3+^ ions at the workpiece convex point is higher than that at the concave point. Therefore, the material removal rate at the workpiece convex point is faster, which realizes the flattening of the workpiece surface [22,23,25].

**Figure 2:**
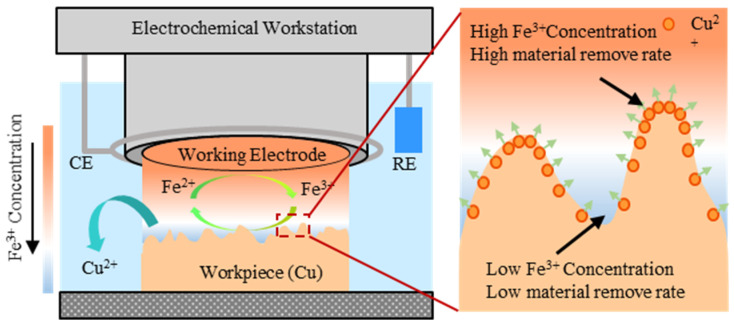
The schematic of EGCP.

The whole process can be represented as follows (Figure 2):

1. The electrochemical reaction to generate the Fe^3+^ ion near the WE:

(5) $${\mathrm{Fe}}^{2+}\to {\mathrm{Fe}}^{3+}+e^{-}$$

where Fe^2+^ ion is the intermediary, Fe^3+^ ion is the etching agent and e^−^ is an electron.

2. The chemical etching reaction between the Cu atom and Fe^3+^ ion:

(6) $${2\mathrm{Fe}}^{3+}+\mathrm{Cu}\to 2{\mathrm{Fe}}^{2+}+{\mathrm{Cu}}^{2+}$$

When using EGCP to process an ultra-flat surface, it is necessary to prepare a WE whose shape accuracy is much higher than that of the target workpiece. This demand increases the manufacturing cost and manufacturing difficulty of the WE. The dynamic etching of EGCP keeps the WE rotation at a constant speed during processing. In this way, the profiles of the WE are homogenized, which is equivalent to improving the shape accuracy of the WE. This allows better shape accuracy without increasing the manufacturing difficulty of the WE. However, the mass transfer of the ions in the electrolyte must be controlled by diffusion, which realizes the flattening of the workpiece.

**Figure 3:**
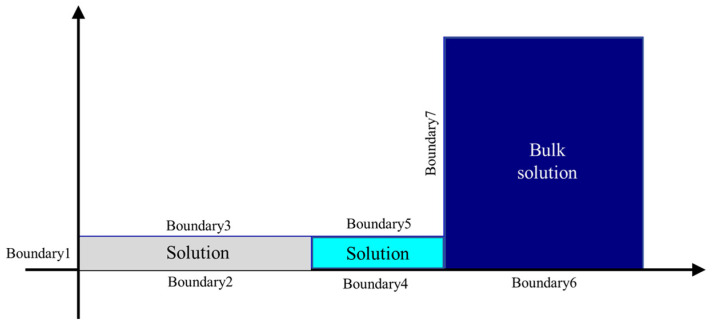
Convection–diffusion model of the EGCP.

In our previous research, Shan et al. [22,23] established a theoretical model to simulate the EGCP process, which was verified experimentally. Based on the model and governing equations reported in the literature, an improved model was established by COMSOL Multiphysics (version 6.3, COMSOL Inc., Stockholm, Sweden). This model can be used to investigate the influence of WE rotation on etching agent (Fe^3+^) distribution by adjusting the rotational speeds of boundaries 3, 5, and 7 (0, 0.1 rad/s, and 1.2 rad/s) in the laminar flow physics interface with the rotating wall condition. This model includes three zones: the bulk solution zone, the insulating layer covering zone (under boundary 5), and the etching zone (under boundary 3) (Figure 3). The etching agent Fe^3+^ is mainly distributed under boundary 3. The function of the insulating layer (boundary 5) is to prevent the etching agent Fe^3+^ moving into the bulk solution. The establishment of this model has the following assumptions.

1. The electrolyte is incompressible.
2. For simplicity, only the concentration distribution of the etching agent at the junction of the WE, the insulation layer, and the bulk solution is considered.
3. The effects of the concentration of Cu^2+^ ions on the etching behavior of Cu in EGCP are ignored.

**Figure 4:**
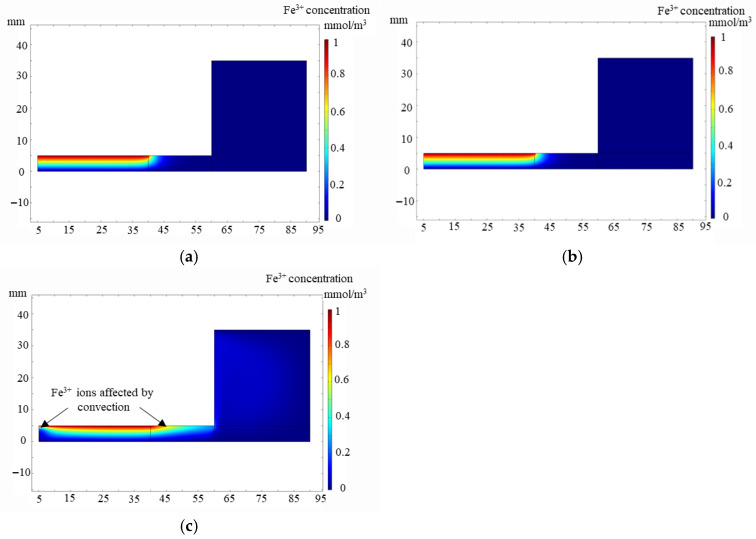
Influence of WE rotation speeds on the distribution of the etching agent in EGCP. (**a**) *w* = 0 rad/s; (**b**) *w* = 0.1 rad/s; (**c**) *w* = 1.2 rad/s.

As is displayed in Figure 4, when the WE remains static (*w* = 0 rad/s), the mass transfer of ions in the electrolyte only relies on diffusion. As can be seen in Figure 4a, the concentration distribution of the etching agent Fe^3+^ ions presents a gradient distribution in the Z-direction. The concentration of Fe^3+^ ions near the WE surface is much higher than the concentration of Fe^3+^ ions near the workpiece surface (boundary 2). At this time, the mass transfer of the ions in the electrolyte is controlled by diffusion and EGCP can achieve a good flattening effect. This is proved by the previous analysis and experiments [22,23,24,25]. When the WE rotation speed increases to 0.1 rad/s, the WE rotates slowly. The rotation of the WE does not break the concentration distribution in the Z-direction (Figure 4b); the concentration of Fe^3+^ ions still presents a gradient distribution, meeting the necessary conditions for flattening. When the WE rotation speed increases to 1.2 rad/s, the concentration distribution of the Fe^3+^ ions under the WE is affected by convection (Figure 4c). From the simulation results, it can be seen that with the increase in the WE rotation speed, the convection effects increase, and the concentration distribution of the etching agent is affected by convection, which destroys the necessary condition for flattening in EGCP. Combined with motor parameters, it is determined that the rotation speed of the WE is 0.02 rad/s to ensure the homogenization effect without affecting the flattening effects in EGCP.

## 3. Experiments and Methodology

### 3.1. The Equipment of the Jet-ECM

**Figure 5:**
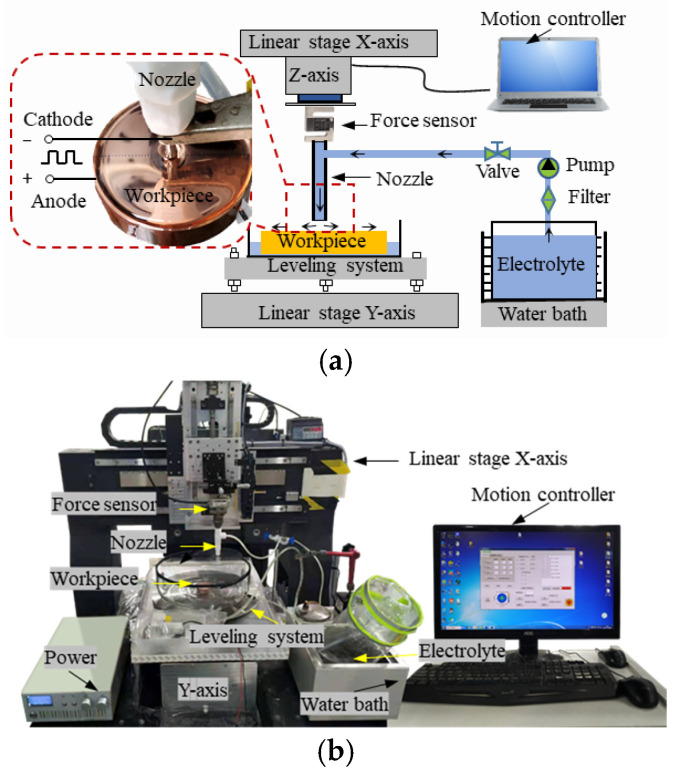
The Jet-ECM system. (**a**) Schematic diagram; (**b**) devices used in Jet-ECM.

The equipment of the Jet-ECM was self-assembled, which can be seen in Figure 5. The whole system includes a motion platform, an electrolyte supply system, a gap control system, and a power supply. The power supply (NMIDZ220AD24V10A, Suzhou Nine-Dream Information Technology Co., Ltd., Suzhou, China) supports both direct current and pulse modes, with adjustable duty cycles ranging from 0% to 100%. The motion platform (Beijing U-Precision Tech Co., Ltd., Beijing, China) has a repetitive positioning accuracy of 0.2 μm (X-axis) and 0.5 μm (Y-axis). The workpiece is placed in the cell without clamping. The nozzle is driven by the Z-direction and the X-direction guide rails, and the cell can be driven by the Y-direction guide rail. The force sensor is used to judge the contact state between the nozzle and the workpiece. When the nozzle contacts the workpiece, the force sensor indicates the force change. At this time, the nozzle is in contact with the workpiece, which indicates the gap between the nozzle and the workpiece is zero. Then, the nozzle is moved away from the workpiece through the control system to the specified distance. The leveling system is used to ensure the workpiece is level.

The workpiece is connected to the anode of the power, and the nozzle is connected to the cathode of the power. A nickel nozzle with an inner diameter of 1 mm is selected, and the workpiece is made of oxygen-free Cu with a diameter of φ 50 mm. A specific acid electrolyte based on phosphoric acid is used, which was validated in our previous research [18]. The specific composition of the electrolyte is shown in Table 1. The electrolyte is stored in a water bath with a temperature of 35 °C. A pump is used to eject the electrolyte onto the workpiece to form a stable hydraulic jump on the workpiece surface. To prevent equipment corrosion, all wetted components are made of acid-resistant materials.

The power supply can provide pulse power and adjust the duty ratio (0–100%). The processing parameters are shown in Table 2.

### 3.2. The Equipment of EGCP

**Figure 6:**
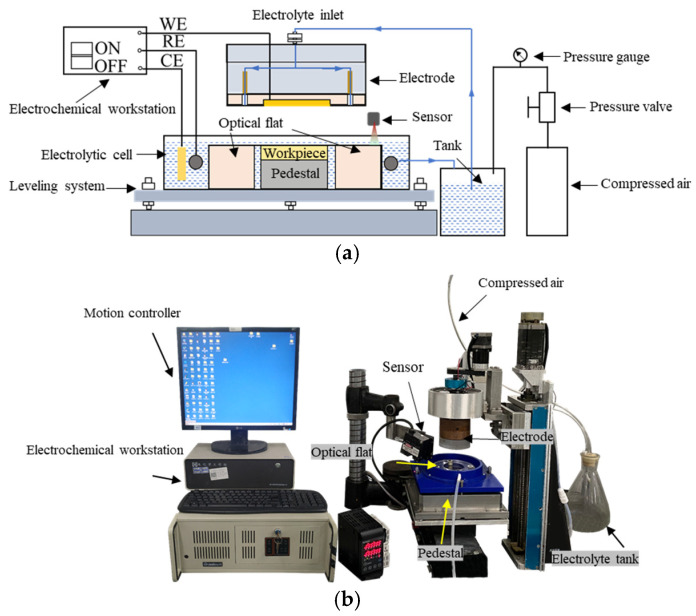
The EGCP system. (**a**) Schematic diagram; (**b**) devices used in EGCP.

As shown in Figure 6, the EGCP setup was also self-assembled and includes an electrolyte supply system, a motion system, a leveling system, an electrochemical workstation and an ultra-flat WE. The electrochemical reaction is controlled by the electrochemical workstation (CHI760E, Shanghai Chenhua Instruments Co., Ltd., Shanghai, China). Different from Jet-ECM, the electrolyte supply system used the principle of hydrostatic support to control the gap between the WE and the workpiece. The electrolyte supply system includes a compressor, a pressure valve, a pressure gauge, and a tank. The compressed air is generated by the compressor and adjusted by the pressure valve. Then, the air is pumped into the tank. The polishing electrolyte is stored in the tank and pumped using compressed air. The electrolyte passes through the WE and is ejected onto the flat crystal. Based on the principle of hydrostatic support, a thin liquid film is generated between the WE and the flat crystal. By adjusting the air pressure, the flow rate of the electrolyte can be controlled to adjust the thickness of the liquid film. The thickness of the liquid film can be used to indicate the machining gap. The motion system realizes the WE rotation through a motor. The straight bearings and bellows are used to ensure the stability of the WE rotation. The leveling system includes a sensor and three adjusting screws. The role of the leveling system is to ensure that the workpiece top surface and the flat crystal top surface are located on the same plane.

**Figure 7:**
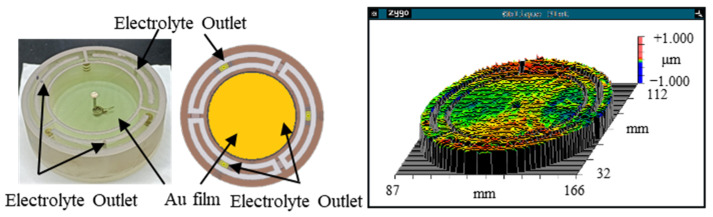
The shape accuracy of the Au WE (PV = 535 nm).

The WE is made of ultra-flat quartz glass to guarantee shape accuracy. To guarantee electrical conductivity, the WE is covered by a film of gold. The flatness of the electrode is 535 nm, which is measured by Flat Master 200 (Corning Incorporated, Corning, NY, USA), as shown in Figure 7.

The specific composition of the electrolyte for EGCP is shown in Table 3. In EGCP, a three-electrode system is used. A platinum ring and a saturated mercury sulfate electrode (MSE) are used as the counter electrode (CE) and the reference electrode (RE), respectively. The machining voltage of 0.35 V (vs. MSE) is provided by the electrochemical workstation CHI760E (Shanghai Chenhua Instruments Limited., Shanghai, China). The gap between the WE and the workpiece is maintained at 25 μm, and the WE rotation speed is 0.02 rad/s. The WE is lifted with a fixed time (20 min) to ensure the stability of the ion concentration in the micro gap.

## 4. Results and Discussions

### 4.1. Parameters for Jet-ECM

To study the effects of voltages in Jet-ECM, voltages of 1.5 V (in the passivation zone) and 3.5 V, 6.5 V, and 10 V (in the over-passivation zone) were selected for processing, based on the polarization curve of pure Cu in the specific acid electrolyte [18]. The other parameters were a pulse frequency of 10 kHz, a duty cycle of 50%, a processing time of 180 s, and an electrolyte flow rate of 300 mL/min.

**Figure 8:**
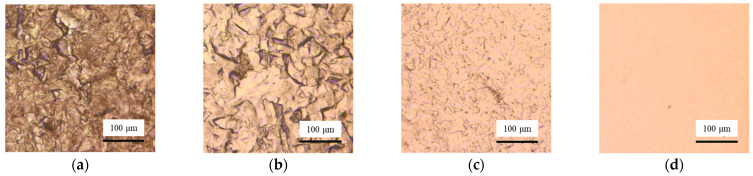
Effect of voltages on the surface quality of Jet-ECM. (**a**) 1.5 V; (**b**) 3.5 V; (**c**) 6.5 V; (**d**) 10 V.

As shown in Figure 8, lots of grain boundaries and defects can be seen on the surface after machining with 1.5 V voltage. With the increase in the voltage, the surface defects and grain boundaries are reduced significantly. When the voltage is 10 V, the surface after processing is smooth. The currents are recorded during processing with a current sensor CP5030 (SIGLENT Technologies Co., Ltd., Shenzhen, China). When the voltages are 1.5 V, 3.5 V, 6.5 V, and 10 V, the currents are 4.5 mA, 28.6 mA, 64.7 mA, and 82.9 mA, respectively. In electrochemical machining, the high current density is conducive to improving the surface quality. When the current density is low, the mass transfer rate of ions to the bulk solution is faster than the generating rate of Cu ions. The viscous film cannot form on the workpiece surface. With the increase in the current, more Cu ions are generated, and the viscous film is gradually formed to improve the surface quality. In addition, it is found that compared with static electrochemical etching, Jet-ECM processing requires a higher voltage to generate the viscous film. In Jet-ECM, the flow rate of the electrolyte is high, which enhances the mass transfer rate through convection and increases the formation difficulty of the viscous film.

**Figure 9:**
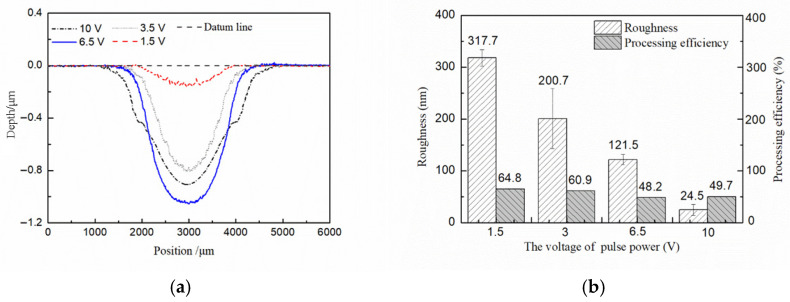
The effects of voltages on profiles in Jet-ECM. (**a**) Profiles per second with different voltages; (**b**) roughness and processing efficiency.

Different from the principle of surface roughness, the material removal rate increases first and then decreases with the increase in voltages. When the voltage is lower than 6.5 V, the material removal rate increases with the increasing voltages. However, when the voltage increases to 10 V, the material removal rate decreases, and the radial material resolution is significantly decreased (Figure 9a). This is because the radial material removal resolution is affected by the double electric layer on the workpiece surface. Increasing the voltage expands the domain of the workpiece achieving the decomposition potential, which significantly decreases the radial material resolution. In addition, the formation of the viscous film and the increase in the side reactions caused by excessive voltage are also the reasons for the reduction in the processing efficiency (Figure 9b). On this basis, 6.5 V is chosen as the optimal voltage.

**Figure 10:**
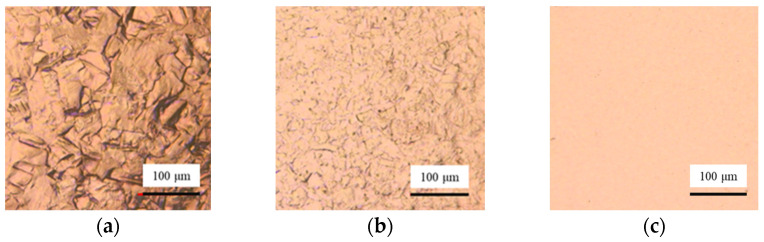
Influences of duty ratio on surface quality in Jet-ECM. (**a**) 25%; (**b**) 50%; (**c**) 75%.

**Figure 11:**
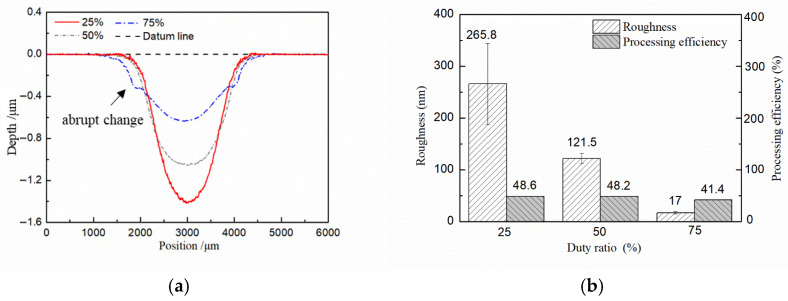
Effects of duty ratio in Jet-ECM. (**a**) Profiles per second with different duty ratios; (**b**) roughness and processing efficiency.

To study the rules of duty ratio on the surface quality and machining accuracy in Jet-ECM, the duty ratios of 75%, 50%, and 25% are used for testing. The other parameters are a voltage of 6.5 V, a pulse frequency of 10 kHz, and an electrolyte flow rate of 300 mL/min. The results show that the surface quality is improved with the increasing value of duty ratio (Figure 10 and Figure 11b). This identifies that the surface quality is not only affected by voltages and currents but also can be improved by increasing the duty ratio. When a 25% duty ratio is adopted, the machined Cu surface has obvious grain boundaries and defects. When 50% and 75% duty ratios are adopted, the machined surface quality is significantly improved compared to the 25% duty ratio. However, when adopting a 75% duty ratio, the contour edge has abrupt changes (Figure 11a). Considering both the accuracy and the surface quality, the machining parameters of Jet-ECM are finally determined as 6.5 V, 10 kHz, and a 50% duty ratio.

**Figure 12:**
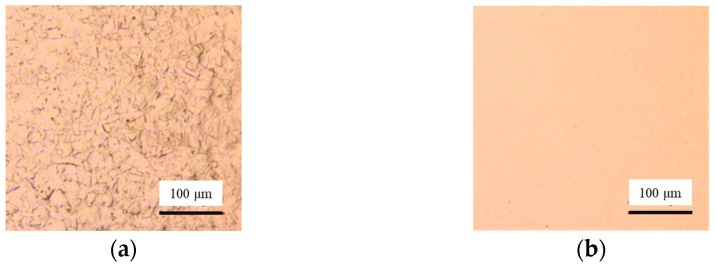
The influence of electrolyte flow rate on the surface quality. (**a**) 460 mL/min; (**b**) 300 mL/min.

However, some defects can still be seen on the workpiece surface under these processing parameters (Figure 10b). According to Equation (3), the flow rate of electrolytes has an important effect on the viscous film. To further improve the surface quality, the electrolyte flow rate is reduced to promote the formation of the viscous film, thus improving the surface quality. As shown in Figure 12, the electrolyte flow rate decreases from 460 mL/min to 300 mL/min. The other parameters are a voltage of 6.5 V, a pulse frequency of 10 kHz, and a duty cycle of 50%. The surface roughness decreases from 121.5 nm to 40.5 nm. Finally, the parameters are determined as 6.5 V, 10 kHz, a 50% duty ratio, and an electrolyte flow rate of 300 mL/min.

### 4.2. The Stability of Jet-ECM

High stability is an important factor for Jet-ECM to be used in CCOS. The optimal parameters of Jet-ECM selected are used to process grooves, and the groove morphology could be used to analyze the stability. The stability here refers to the consistency of the groove profiles at different positions. The fixed nozzle moving speeds of the nozzle were set to 0.25 mm/s and 0.5 mm/s, the radii of the revolution were set to 7 mm and 14 mm, and the numbers of revolutions were set to 5 and 10, respectively. Three cross-section profiles were chosen at different positions of the groove for comparison.

**Figure 13:**
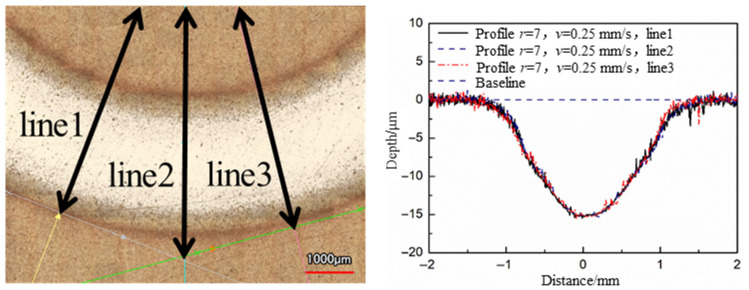
The profile stability of the groove with *r* = 7 mm, *v* = 0.25 mm/s, and 5 turns.

**Figure 14:**
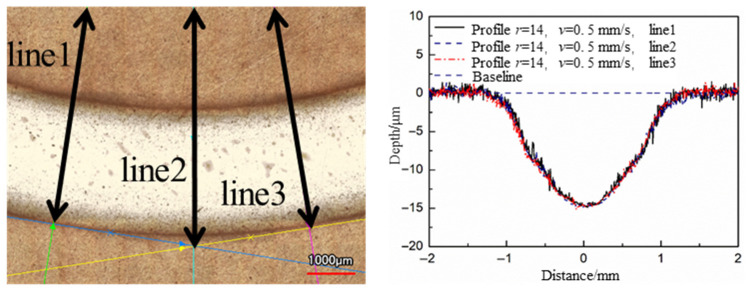
The profile stability of the groove with *r* = 14 mm, *v* = 0.5 mm/s, and 10 turns.

The 3D morphology and cross-sectional profiles of the grooves were measured by a laser confocal microscope (VK-X250, Keyence Co., Osaka, Japan). The results show that the cross-section profiles at different positions of the same groove have a high coincidence, which verified the high stability of the Jet-ECM with the optimal parameters selected (Figure 13 and Figure 14). According to Faraday’s Law, the anodic dissolution rate of Cu is correlated with the current value, and the stability of the currents is another important factor to evaluate the stability of the process. During processing, the currents were recorded with a current sensor CP5030 (SIGLENT Technologies Co., Ltd., Shenzhen, China), and the currents were randomly measured as 29.22 mA and 28.88 mA, respectively. The error was less than 1%.

### 4.3. Correcting the Surface Shape Error with the Two-Step Etching Process Chain

Firstly, the Jet-ECM is applied to the CCOS, which uses electrochemical etching to achieve the rapid convergence of the shape error. By processing multiple ring grooves on the workpiece surface by Jet-ECM, the peak region of the workpiece superimposes deep ring grooves, and the valley region of the workpiece superimposes shallow ring grooves. In this way, the shape error of the workpiece is corrected.

**Figure 15:**
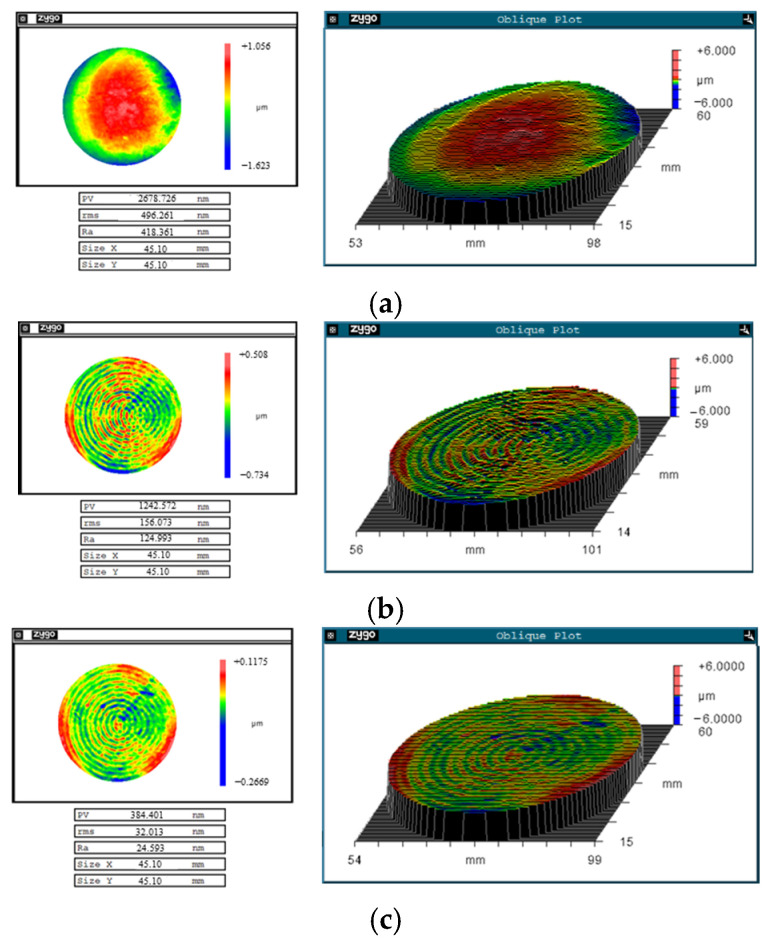
The shape accuracy of the Cu workpiece. (**a**) Initial PV 2.678 μm; (**b**) after computer-controlled optical surfacing PV 1.242 μm; (**c**) after EGCP PV 0.384 μm.

The 3D morphology of the initial workpiece surface was measured. As shown in Figure 15a, the PV value of the workpiece is 2.678 μm. The CCOS parameters such as nozzle trajectories, nozzle moving speeds, and rotation numbers were established by the model and setups established by the author earlier [18,31]. After 34 min of processing, the PV value of the φ 50 mm workpiece (valid dimensions = 90% of the central region) is reduced from 2.678 μm to 1.242 μm, as shown in Figure 15a,b. The shape error of the workpiece surface is corrected and the bulge of the workpiece is completely removed. However, the nozzle size (inside diameter 1 mm) is much smaller than the workpiece size (50 mm), which leads to the MSF error with an error period between 0.08 mm and 3 mm [31]. The EGCP was used as the follow-up process after CCOS to further correct the surface shape error of the workpiece and reduce the MSF error caused by CCOS.

In EGCP, the electrochemical workstation provides a voltage of 0.35 V (vs. MSE). The rotation speed of the WE is 0.02 rad/s. The WE is lifted with a fixed time (20 min) to maintain the concentration of Cu ions in the micro gap. As shown in Figure 15c, after 2.5 h of polishing, the surface PV value of the workpiece is reduced to 0.384 μm.

**Figure 16:**
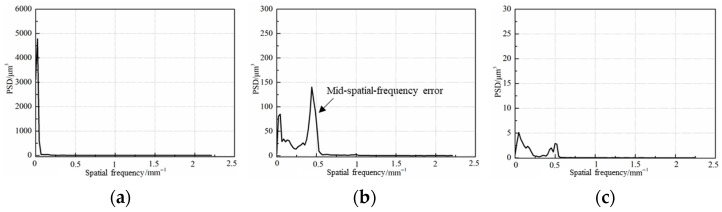
MSF error analysis. (**a**) Initial; (**b**) after computer-controlled optical surfacing; (**c**) after EGCP.

The power spectral density (PSD) is used to evaluate the MSF error of the workpiece, which can show the spatial frequency distribution of the PSD. The 3D morphology measured by FlatMaster200 (Corning Company, NY, USA) (Figure 15) and 20 line profiles (uniform angular sampling) are selected for PSD calculation, to make a more accurate assessment. After the computer-controlled deterministic polishing, the surface shape accuracy is rapidly improved. However, the MSF error rises significantly (Figure 16b) and the MSF error is significantly reduced after full-diameter polishing by EGCP (Figure 16c).

## 5. Conclusions

In this paper, a stress-free etching process chain is proposed, which can correct the surface shape error efficiently. According to the results, the following conclusions are drawn.

(1) A new two-step stress-free polishing process chain is proposed, which uses controllable electrochemical etching and chemical etching to correct the surface shape error of the Cu surface. The Jet-ECM, which uses the electrochemical etching mechanism, can be applied to the computer-controlled optical surfacing to correct the shape error of the workpiece with high efficiency. In addition, EGCP, which uses the mechanism of diffusion-controlled chemical etching, is implemented as a follow-up process to reduce the MSF error caused by computer-controlled optical surfacing. During the whole process, the workpiece does not contact any tool. In addition, the workpiece does not need to be clamped, which is helpful to obtain a substrate with high shape accuracy.
(2) According to the theory of viscous film, increasing the voltage and duty ratio are conducive to the formation of the viscous film in Jet-ECM. After the formation of the viscous film, the surface quality and the stability of electrochemical machining can be improved significantly. Increasing the electrolyte flow rate increases the effect of convection, which is not conducive to surface quality in Jet-ECM.
(3) The dynamic etching of EGCP keeps the WE rotation at a constant slow speed during processing. In this way, the profiles of the WE are homogenized, which is equivalent to improving the shape accuracy of the WE. Through this process, better shape accuracy can be obtained without increasing the cost and manufacturing difficulty of the WE.

Several limitations still need further exploration. Currently, workpiece profile measurements rely on offline detection, which introduces extra positioning errors and increases operational complexity. Future work will develop an automated deterministic polishing system integrated with online profile measurement to minimize error sources. And, orthogonal experiments will be carried out to further optimize the CCOS processing parameters.

## Figures and Tables

**Table 1:** Composition and proportion of electrolyte.

Phosphoric Acid	Ethanol	Lactic Acid	Benzotriazole	Ammonium Acetate
850 mL	90 mL	60 mL	6 g	3 g

**Table 2:** Jet-ECM processing parameters.

Composition	Value
Voltage/V	1.5, 3.5, 6.5, 10
Frequency/kHz	10
Duty ratio/%	25, 50, 75
Gap/μm	600
Electrolyte temperature/°C	35
Electrolyte flow rate/mL/min	460, 300

**Table 3:** The composition of the electrolyte in EGCP.

Composition	Value
FeSO_4_/mmol/L	1
H_2_SO_4_/mol/L	0.2
Benzotriazole/mmol/L	1

## Data Availability

The original contributions presented in this study are included in the article. Further inquiries can be directed to the corresponding author.
